# Effects of short bout small-sided game training on acid-base balance markers in youth male soccer players

**DOI:** 10.1038/s41598-023-30646-4

**Published:** 2023-03-02

**Authors:** Jakub Kryściak, Tomasz Podgórski, Paweł Chmura, Marek Konefał, Jan Chmura, Marius Brazaitis, Toni Modric, Marcin Andrzejewski

**Affiliations:** 1 Department of Physiology and Biochemistry, Poznań University of Physical Education, 61-871 Poznan, Poland; 2 Department of Team Games, Wroclaw University of Health and Sport Sciences, 51-612 Wrocław, Poland; 3 Department of Biological and Motor Sport Bases, Wroclaw University of Health and Sport Sciences, 51-612 Wrocław, Poland; 4 Institute of Sports Science and Innovation, Lithuanian Sports University, 44221 Kaunas, Lithuania; 5 Faculty of Kinesiology, University of Split, 21000 Split, Croatia; 6 Department of Methodology of Recreation, Poznań University of Physical Education, 61-871 Poznan, Poland

**Keywords:** Biochemistry, Physiology

## Abstract

This study aimed to compare the effects of 1 × 1 small-sided games (SSGs) with different bout durations on external (ETL) and internal training loads (ITL) in youth soccer players. Twenty U18 players were divided into two groups performing six 1 × 1 SSGs with 30 and 45 s bout durations on a playing field of 10 by 15 m. ITL indices, including the percentage of maximum heart rate (HR), blood lactate (BLa) level, pH, bicarbonate (HCO_3_^−^) level, and base excess (BE) level, were measured at rest, after each SSG bout, and 15 and 30 min after the entire exercise protocol. ETL (Global Positioning System metrics) was recorded during all six SSG bouts. The analysis showed that the 45 s SSGs had a greater volume (large effect) but a lower training intensity (small to large effect) than the 30 s SSGs. A significant time effect (*p* < 0.05) was observed in all ITL indices and a significant group effect (F_1, 18_ = 8.84, *p* = 0.0082, ƞ^2^ = 0.33) in the HCO_3_^−^ level only. Finally, the changes in the HR and HCO_3_^−^ level were smaller in the 45 s SSGs than in the 30 s SSGs. In conclusion, 30-s games, characterized by a higher intensity of training effort, are more physiologically demanding than 45-s games. Secondly during short-bout SSG training the HR and BLa level have limited diagnostic value for ITL. Extending ITL monitoring using other indicators, such as the HCO_3_^−^ and BE levels, appears reasonable.

## Introduction

Soccer is a team sport characterized by a high-intensity intermittent activity profile with metabolic contributions from both the aerobic and anaerobic energy pathways^1–3^. Recently, it has become a more intensified and physically demanding game, in which players need to cover about 30% longer distances above the high-intensity threshold^4^. Accordingly, soccer players should have a high fitness profile to cope with the expected intensified demands of a match^4,5^. Although soccer players need to have a multifactorial fitness status to perform better in a match^6^, high-intensity intermittent fitness is crucial because it improves performance^7^, shortens the recovery time after a match^8^, and reduces the risk of injury^9^.

Training stimuli should be similar to those of the competitive demands of soccer to achieve maximum adaptation to high-intensity intermittent physical loads^10^. A common method used to address this need is game-based training with particular emphasis on small-sided games (SSGs)^11–15^. SSGs have a comparable structure to the real game but are played in small pitches and have modified rules^11,16^; their intensity can be easily modified by several factors, including bout duration^17,18^, number of players^19,20^, and pitch dimensions^19,21,22^. All of these organizational variables have a key impact on the internal (ITL) and external training loads (ETL) of athletes^23^.

In SSGs, the global positioning system (GPS) is used to monitor the ETL, defined as the work conducted by athletes^24–26^. In running-based team sports, such as soccer, the GPS is a valid and reliable tool that can measure the activity profiles of athletes, including speed, time spent in different speed zones, total distance (TD) covered, distance covered in different speed zones, player load, and number of direction changes, accelerations, and decelerations^27,28^. The ETL is inextricably linked with and influences the ITL, defined as the body's response to biological stressors imposed on athletes during training and competition exercise. Subjective methods, such as evaluation of the rate of perceived exertion (RPE)^11,17,29,30^, or objective methods, such as measurement of the heart rate (HR) or blood lactate (BLa) level, are used to quantify the ITL^16,18,29,31^. Previous studies have also shown relationships between the ETL measured using the GPS and ITL indices, such as the HR, RPE, or BLa level^32–35^.

Lactate is an end-product of anaerobic glycolysis and is often used to evaluate the intensity of a match or training effort. Bla levels between 4 and 13 mmol/L after SSGs^18,29,31,36,37^ indicate a substantial involvement of anaerobic metabolism and the possibility of the application of SSG training to the development of soccer-specific speed endurance capabilities^31,36,38^. Similar BLa levels have also been observed during and after simulated^39^ and actual^40,41^ match plays, which are related to high-intensity activities, including sprints, changes of direction, accelerations, decelerations, and technical actions.

The common judgment that an increased post-exercise BLa level alone causes fatigue can be misleading. The inevitable consequence of energy production during glycolysis is not only the production of lactate but also the release of hydrogen ions (H^+^)^42,43^. Consequentially, hydrogen ion accumulation and intramuscular pH reduction could influence exercise performance because the enzymes involved in glycolysis and oxidative phosphorylation are inhibited; the calcium ions (Ca^2+^) binding to troponin C are reduced; and the sarcoplasmic reticulum enzyme Ca^2+^ ATPase is inhibited^42,44^. Despite the discussion on the effects of glycolytic metabolism on fatigue^43^, exercise-induced pH reduction and acid–base balance (ABB) disturbances are one of the potential triggers of fatigue in soccer^1,45,46^. The free H^+^ released during glycolysis can be buffered by bicarbonate (HCO_3_^−^), causing the nonmetabolic production of carbon dioxide (CO_2_) and consequently increasing the rate of ventilation^47,48^. Therefore, acid–base homeostasis is an important part of exercise capacity in soccer^1,45,46^.

Accordingly, a specific training tool that substantially induces glycolytic metabolism, increases the ability to maintain ABB, and specifically improves the speed endurance of soccer players is short-bout 1 × 1 SSGs^18^. These SSGs improve technical skills, including skills required during one-on-one duels, which play an increasingly important role in the final score of modern soccer^49^. Most scientific studies on soccer have focused on ABB merely based on changes in the BLa level^18,29,31,36,40,41^. Few studies^39,50–53^ have analyzed changes in ABB indicators, such as the pH, base excess (BE) level, or HCO_3_^−^ level, in relation to the BLa level; no study has yet evaluated such changes in SSGs. In addition, previous works on the relationship between the ETL and ITL have based their evaluation on GPS readings and HR, RPE, or BLa level, respectively^32–35^. No study has yet evaluated the relationship between the activity profiles of athletes (e.g., maximum velocity [Vmax], player load, and distance covered) and blood ABB markers (e.g., pH, BE level, or HCO_3_^−^ level). We are convinced that our research will provide new knowledge on the impact of short bout SSGs training on the ABB homeostasis, and thus will complement the existing methods of ITL monitoring in youth soccer training. Therefore, the primary aim of our study was to compare the potential effects of 1 × 1 SSGs with different bout durations (30 and 45 s) but with the same work-to-rest ratio (1:4) on the ETL (GPS metrics) and ITL (HR, BLa level, pH, BE level, and HCO_3_^−^ level) of youth soccer players. The secondary aim was to determine the potential relationships between the ETL and ITL indices.

We hypothesize that the different bout duration of the SSGs used will affect both the intensity and the volume of the training, and thus the physiological changes caused by these SSGs will be different.

## Methods

### Study design

The primary aim of the research was to determine the effects of repeated 1 × 1 SSGs (Table 1) with different bout durations (30 s: group E1, 45 s: group E2) but with the same work-to-rest ratio (1:4) on the ETL (GPS metrics) and ITL (BLa level, blood ABB parameters, and percentage of maximum heart rate [%HRmax]) in youth soccer players. For this purpose, 20 youth soccer players were randomly divided into two groups participating in six repeated 1 × 1 SSGs.

**Table 1:** Characteristics of small-sided games used during the study.

1 × 1 SSG group	Bout duration (s)	Number of bouts	Rest between bouts (s)	Exercise to rest ratio	Pitch dimensions W × L (m)
E1	30	6	120	1:4	10 × 15
E2	45	6	180	1:4	10 × 15

SSG—small-sided game, W × L—width and length, E1—6 × 30 s 1 × 1 SSG, E2—6 × 45 s 1 × 1 SSG.

**Figure 1:**
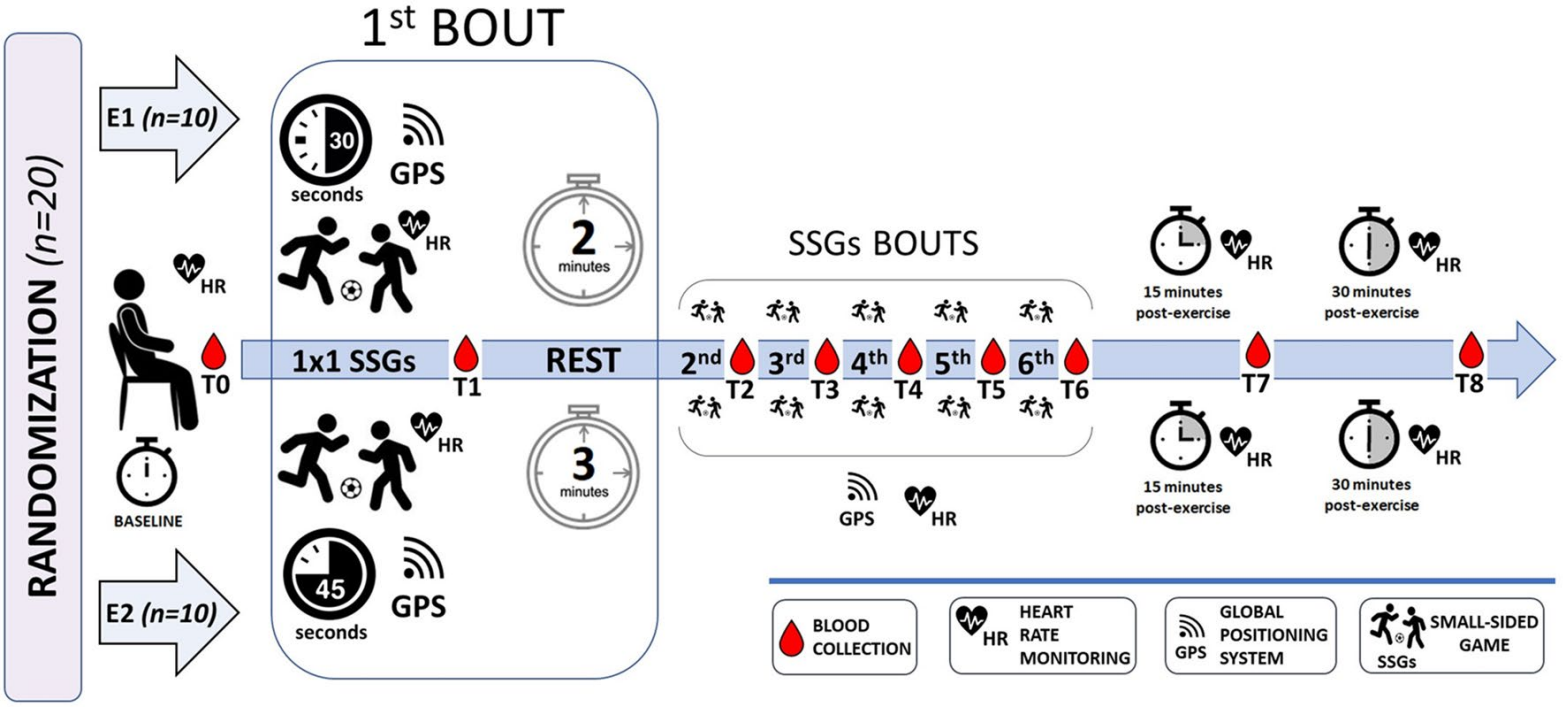
Experimental protocol. SSGs—small–sided games; E1—6 × 30 s 1 × 1 SSGs; E2—6 × 45 s 1 × 1 SSGs; HR—heart rate; GPS—global positioning system.

To measure the BLa level and ABB parameters, blood samples were obtained nine times from each athlete: at rest (T0), after each of the six SSG bouts (T1–T6), and 15 and 30 min after the entire exercise protocol (T7 and T8, respectively) (Fig. 1). Between each bout, the players recovered passively by sitting.

The HR was continuously measured from the beginning to the end of the study protocol using short-range radio telemetry (Polar Sport Tester, Polar Electro Oy, Kempele, Finland). For the analysis, the average 5 s values from the same measurement points as in blood sampling were considered.

The athletes’ activity profiles during the SSGs were measured using a portable GPS (Catapult S5 Melbourne, Innovations, Australia).

### Participants

Twenty U18 outfield male soccer players belonging to the same academy team from a first-division club in Poland participated in the study. The mean values of body height and body mass measured in both groups of players (body height: E1 = 177.9 ± 5.08, E2 = 178.4 ± 4.92; body mass: E1 = 70.5 ± 5.55, E2 = 71.2 ± 5.31; respectively) did not differ statistically significantly (Table 2). Similarly, the lack of statistically significant differences between the groups was demonstrated in the case of the mean values of age and training experience (age: E1 = 17.2 ± 0.88, E2 = 17.1 ± 0.75; training experience E1 = 8.6 ± 3.35, E2 = 8.7 ± 3.15; respectively).

**Table 2:** Anthropometric characteristics and training experience of youth soccer players (mean ± SD).

Variable	Group E1 (n = 10)	Group E2 (n = 10)	t Value	*p* Value
Age (years)	17.2 ± 0.88	17.1 ± 0.75	0.2735	*p* ≥ 0.05
Body height (cm)	177.9 ± 5.08	178.4 ± 4.92	0.2236	*p* ≥ 0.05
Body mass (kg)	70.5 ± 5.55	71.2 ± 5.31	0.2882	*p* ≥ 0.05
Training experience (years)	8.6 ± 3.35	8.7 ± 3.15	0.0688	*p* ≥ 0.05

E1—6 × 30 s 1 × 1 SSGs, E2—6 × 45 s 1 × 1 SSGs.

In every competitive microcycle, the players trained five times on average and played one official match at the highest level in their age category in Poland. The exclusion criterion was the competitor's failure to complete the research protocol or failure to obtain an appropriate blood sample necessary to perform the biochemical analysis. The data obtained from all 20 participants were considered for further analysis. The participants as well as their parents or legal tutors and coaches were fully informed about the study procedures and the experimental risk. Written, informed consent from all the research participants or legal tutors was obtained. The study was approved by the Research Ethics Committee (no. 20/2017) of the University School of Physical Education in Wroclaw and followed the institutional ethical requirements for human experimentation under the Declaration of Helsinki.

### Procedures

Six bouts of 1 × 1 SSGs were used in the study^54^. The 1:4 work-to-rest ratio used herein is often applied in soccer-specific speed endurance training^19,38^.

The players were randomly divided into two groups (E1, n = 10; E2, n = 10) performing 1 × 1 SSGs with different bout durations. In groups E1 and E2, 30 and 45 s bout durations and 120 and 180 s passive rest intervals were used, respectively. The players were asked to be as committed to the training as possible. Additionally, during the SSGs, verbal encouragements were given by the coaches. The SSGs were conducted on a pitch with a synthetic surface with a dimension of 10 × 15 m surrounded by side-boards. The games were played with small goals but without goalkeepers. To ensure a high tempo, the coach fed balls in to the players as soon as a goal was scored or a ball went out of play. The research protocol was conducted on the same day in the morning (10:00–14:00) in stable weather conditions (sunny weather with almost no wind and an ambient temperature of 10–12 °C). The pitch was dry during all training sessions.

### ITL measurement

The ITL indices during the SSGs included the HR, BLa level, and ABB parameters, such as the pH, BE level, and HCO_3_^−^ level.

All biochemical analyses were performed using capillary blood samples. The samples were obtained by qualified laboratory diagnosticians from the fingertip of the non-dominant hand using a disposable Medlance Red lancet-spike (HTL-Zone, Berlin, Germany) with a 1.5 mm blade and 2.0 mm penetration depth. Blood was collected to heparinized capillary tubes (65 μL), and measurement was performed immediately thereafter. The pH, BE level, HCO_3_^−^ level, and BLa level were determined using a blood gas analyzer (ABL90 FLEX, Radiometer, Copenhagen, Denmark).

The internal intensity was expressed as the HR based on the individual %HRmax. During SSG training and 30 min recovery, the HR was recorded using short-range radio telemetry. Each player’s HRmax was determined using the Yo-Yo Intermittent Recovery Test Level 1 (YYIRTL1)^55,56^ and used as a reference value for the quantification of the HR observed during the SSG protocol. The participants completed the test on the day before the SSG protocol at the same time of day and in similar weather conditions. The validity and reliability of the YYIRTL1 have been described previously^57^. The HR was expressed as the average 5 s values measured after each of the SSG bouts and 15 and 30 min after the end of the sixth game (Fig. 1).

### ETL measurement

The ETL indices during the SSGs included the following: TD covered (m), TD divided by session duration (min) to obtain the intensity value per minute (TD per minute; m), total player load (TPL; a.u), TPL divided by session duration (min) to obtain the intensity value per minute (TPL per minute; a.u), and Vmax (km h^−1^). The indicators of the volume of efforts made during the SSGs were the TD and TPL, and the intensity of the SSGs was evaluated on the basis of the TD per minute, TPL per minute, and Vmax.

The activity profiles during the SSGs were evaluated using the portable GPS with a sampling frequency of 10 Hz and using a 100 Hz triaxial accelerometer. The GPS device was fitted to the upper back of each player using a special harness. It was activated 15 min before the start of the research protocol in accordance with the manufacturer’s instructions. The reliability and validity of the equipment have been previously confirmed^58^.

### Statistical analysis

All statistical analyses were performed using Statistica version 13.0 (StatSoft Inc., Tulsa, OK, USA). All results were reported as means and standard deviations (SDs) calculated using conventional procedures. The normality of each variable was initially tested using the Shapiro–Wilk test, and the coefficients of asymmetry and kurtosis were ascertained. To verify the hypothesis of homogeneity variance, we used Levene’s test.

In the initial stage of statistical analysis, groups E1 and E2 were compared in terms of the ETL indices (i.e., TD, TP, TD per minute, TPL per minute, and Vmax). For normally distributed data, between-group differences were evaluated using an independent samples t-test. For non-normally distributed data, differences were tested using the non-parametric Mann–Whitney U test. To determine the size of the differences between two compared groups according to the ETL variables, we calculated the effect sizes (Cohen’s d) as the difference between the mean values in the groups divided by the pooled standard deviation. Cohen’s d lower than 0.2 was considered irrelevant. An effect size of 0.20–0.50 was considered small; 0.50–0.80, medium; and ≥ 0.80, large^59^.

In the next stage of analysis, the potential impact of bout duration on the dependent ITL variables (i.e., BLa level, pH, BE level, HCO_3_^−^ level, and %HRmax) at various measurement points was assessed. For all these markers, five separate two-way mixed analyses of variance were used to evaluate the effects of the groups (between-subject factor levels: E1 vs. E2) and time (within-subject factor levels: T0–T8) and their interactions. For significant main effects of time and interactions (group and time), Tukey’s HSD post-hoc procedures were used to identify specific differences. For main and interaction effects, a partial eta-square was calculated as a measure of the effect size, which indicates the proportion of variance related to a given effect while controlling for other effects^60^.

In the last stage of analysis, the potential relationships between the independent ETL indices and dependent ITL indices were determined. The correlations between the ETL and ITL variables were evaluated using Pearson correlation coefficients. Statistical significance was set at an alpha value of 0.05 for all statistical procedures.

## Results

### ETL parameters

The mean values of the ETL parameters from all six SSG bouts in both groups are presented in Table 3. The indicators of the volume of efforts made during SSG training, such as the TD (t =  − 16.76, *p* ≤ 0.0001, d = 3.06) and TPL (Z =  − 7.90, *p* ≤ 0.0001, d = 1.78), were significantly lower (large effect) in group E1 than in group E2. The indicators of the intensity of efforts made during the SSGs, such as the distance per minute (t =  − 4.93, *p* ≤ 0.0001, d = 0.90), player load per minute (Z = 4.39, *p* ≤ 0.0001, d = 0.83), and Vmax (t = 2.45, *p* = 0.0160, d = 0.47), were significantly higher (small to large effect) in group E1 than in group E2 (Table 3).

**Table 3:** Mean values of external load markers from six SSG bouts (mean ± SD) measured in both groups of tested youth soccer players.

Variable	E1	E2	t/Z value	*p* Value	Cohen’s d	Effect size
	Mean ± SD	Mean ± SD				
Total distance (m)^a^	70.8 ± 7.54*	96.9 ± 9.37	− 16.76	*p* ≤ 0.0001	d = 3.06	Large
Total player load (a.u)^b^	12.1 ± 1.62*	16.0 ± 2.65	− 7.90	*p* ≤ 0.0001	d = 1.78	Large
Distance per minute (m)^a^	141.6 ± 15.02*	129.2 ± 12.49	− 4.93	*p* ≤ 0.0001	d = 0.90	Large
Player load per minute (a.u)^b^	24.2 ± 3.22*	21.4 ± 3.53	4.39	*p* ≤ 0.0001	d = 0.83	Large
Maximum velocity (km h^−1^)^a^	17.2 ± 1.47*	16.5 ± 1.54	2.45	*p* = 0.0160	d = 0.47	Small

SSGs—small-sided games, E1—6 × 30 s 1 × 1 SSGs, E2—6 × 45 s 1 × 1 SSGs.

*Significantly different from E2, *p* < 0.05.

^a^*t*-test; ^b^Mann–Whitney U test.

### ITL parameters

Table 4 presents the mean (± SD) values of the dependent ITL variables: BLa level, pH, BE level, HCO3^−^ level, and %HRmax for the groups and time points of measurement. In all ITL markers, a significant (*p* < 0.05) time effect was observed. A significant increase in the mean values in each subsequent game (T1–T6) and a gradual decrease in the restitution period (T7 and T8) were found for the BLa level (F_8, 144_ = 162.72, *p* ≤ 0.0001, ƞ^2^ = 0.90) and %HRmax (F_8, 144=_1252.84, *p* ≤ 0.0001, ƞ^2^ = 0.99) (Table 4). Contradicting changes were observed in the ABB parameters: pH (F_8, 144_ = 101.90, *p* ≤ 0.0001, ƞ^2^ = 0.85), BE level (F_8, 144_ = 292.77, *p* ≤ 0.0001, ƞ^2^ = 0.94), and HCO_3_^−^ level (F_8, 144_ = 353.22, *p* ≤ 0.0001, ƞ^2^ = 0.95). Among the ITL indices within 30 min of restitution, only the pH (*p* = 0.1203) returned to the pre-exercise level (T8 vs. T0). The mean values of the remaining indices measured after 30 min of restitution differed significantly from the values measured before the training (BLa level: *p* ≤ 0.0001; BE level: *p* ≤ 0.0001; HCO3^−^ level: *p* ≤ 0.0001; %HRmax: *p* ≤ 0.0001). All post-hoc test values are shown in Table 4.

**Table 4:** Time and group effect for indicators characterizing internal training loads during SSGs in both groups of tested youth soccer players.

Variable	Group		Time point								
			T0	T1	T2	T3	T4	T5	T6	T7	T8
BLa (mMol/L)*	E1	11.4 ± 2.82	1.5 ± 0.18 ^1–8^	8.3 ± 2.72 ^0,2–8^	12.1 ± 3.17 ^0,1,3–6,8^	14.6 ± 3.48 ^0–2,5–8^	16.0 ± 3.50 ^0–2,7,8^	16.7 ± 3.36 ^0–3,7,8^	17.1 ± 3.16 ^0–3,7,8^	10.2 ± 2.85 ^0,1,3–6,8^	6.2 ± 2.00 ^0–7^
	E2	11.3 ± 2.61									
pH*	E1	7.26 ± 0.06	7.40 ± 0.02 ^1–7^	7.30 ± 0.05 ^0,2–6,8^	7.25 ± 0.07 ^0,1,4–8^	7.21 ± 0.08 ^0,1,5–8^	7.19 ± 0.08 ^0–2,7,8^	7.18 ± 0.08 ^0–3,7,8^	7.17 ± 0.08 ^0–3,7,8^	7.30 ± 0.05 ^0,2–6,8^	7.37 ± 0.04 ^1––7^
	E2	7.27 ± 0.06									
BE (mMol/L)*	E1	− 11.0 ± 2.32	1.9 ± 1.32 ^1–8^	− 6.9 ± 2.67 ^0,2–6,8^	− 11.9 ± 2.85 ^0,1,4–8^	− 14.4 ± 3.27 ^0,1,7,8^	− 15.8 ± 3.28 ^0–2,7,8^	− 16.4 ± 3.16 ^0–2,7,8^	− 16.7 ± 3.42 ^0–2,7,8^	− 10.0 ± 3.55 ^0,2–6,8^	− 3.5 ± 2.40 ^0–7^
	E2	− 9.9 ± 3.44									
HCO_3_^−^ (mMol/L)*	E1^#^	15.6 ± 1.14	25.7 ± 1.05 ^1–8^	19.0 ± 1.98 ^0,2–8^	15.4 ± 1.63 ^0,1,3–8^	13.7 ± 1.59 ^0–2,5–8^	13.0 ± 1.53 ^0–2,7,8^	12.5 ± 1.33 ^0–3,7,8^	12.3 ± 1.49 ^0–3,7,8^	16.8 ± 2.17 ^0–6,8^	20.7 ± 2.26 ^0–7^
	E2	17.6 ± 2.20									
%HRmax*	E1	77.2 ± 3.00	39.3 ± 4.39 ^1–8^	87.1 ± 4.35 ^0,7,8^	89.8 ± 2.23 ^0,7,8^	90.0 ± 2.33 ^0,7,8^	90.4 ± 2.17 ^0,7,8^	90.5 ± 1.98 ^0,7,8^	89.8 ± 2.39 ^0,7,8^	57.2 ± 4.02 ^0–6, 8^	52.4 ± 3.22 ^0–7^
	E2	75.4 ± 3.02									

Values characterizing groups and time points are presented as mean ± SD.

E1—6 × 30 s 1 × 1 SSGs; E2—6 × 45 s 1 × 1 SSGs; BLa—blood lactate; pH—blood pH; BE—base excess; HCO_3_^−^—blood bicarbonate; %HRmax—percentage of maximum heart rate; T0—before first SSG, T1—after first SSG; T2—after second SSG; T3—after third SSG; T4—after fourth SSG; T5—after fifth SSG; T6—after sixth SSG; T7—15 min after sixth SSG; T8—30 min after sixth SSG.

*Statistically significant time effect, *p* < 0.05; time effect post-hoc: 0 from T0; 1 from T1; 2 from T2; 3 from T3; 4 from T4; 5 from T5; 6 from T6; 7 from T7; 8 from T8.

^#^Significantly different from E2, *p* < 0.05.

Only the HCO_3_^−^ level significantly (F_1, 18_ = 8.84, *p* = 0.0082, ƞ^2^ = 0.33) differed (by 2 mMol/L on average) between groups E1 and E2. Among the remaining ITL indices, a group effect was not observed (BLa level: F_1, 18_ = 0.01, *p* = 0.9237, ƞ^2^ < 0.01; pH: F_1, 18_ = 0.50, *p* = 0.5033, ƞ^2^ = 0.03; BE level: F_1, 18_ = 089, *p* = 0.3555, ƞ^2^ = 0.05; %HRmax: F_1, 18_ = 3.85, *p* = 0.0653, ƞ^2^ = 0.18) (Table 4).

**Figure 2:**
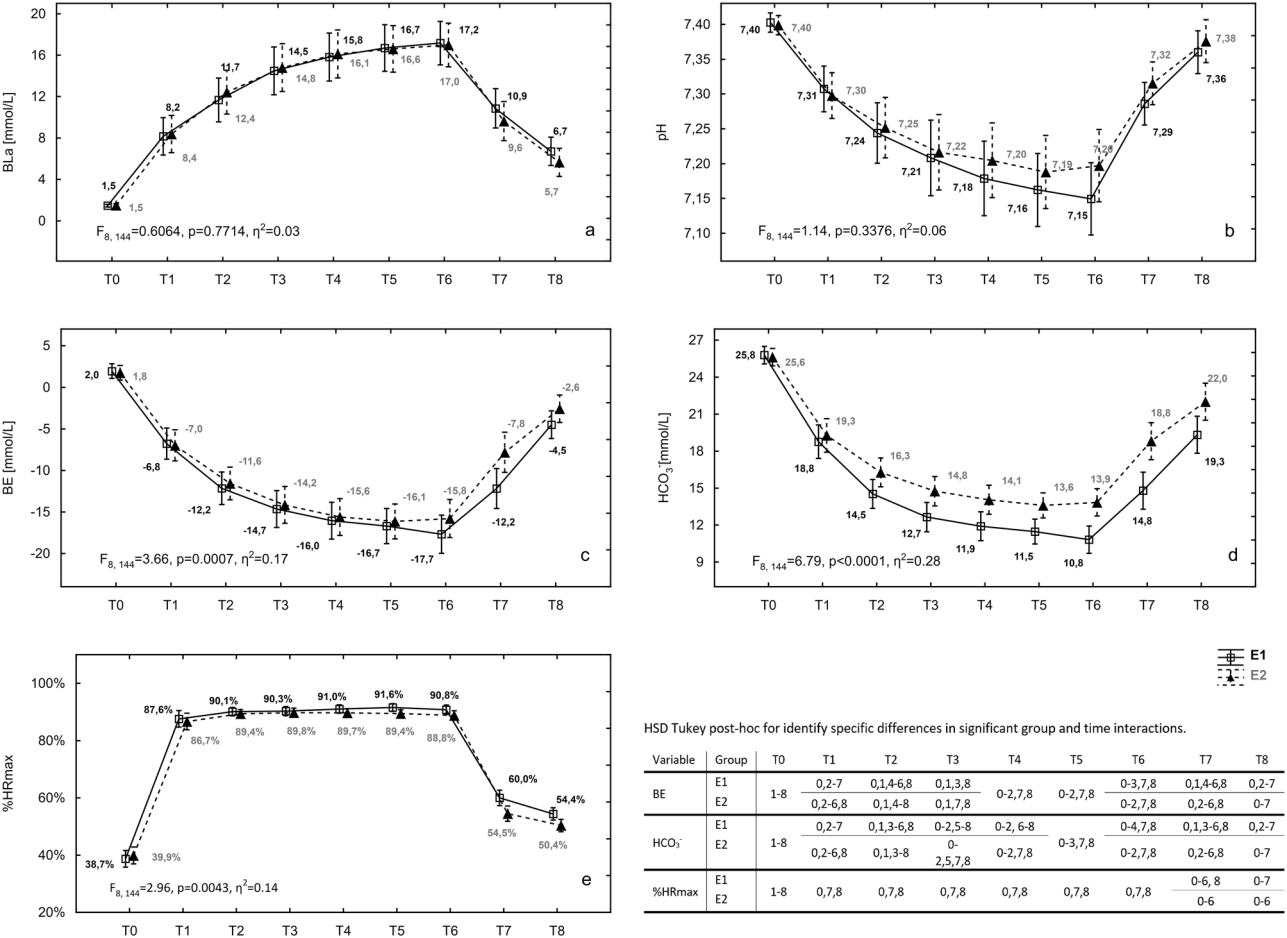
Interaction of the group and time effect for ITL variables with their specific differences (HSD Tukey post-hoc). E1—6 × 30 s 1 × 1 SSGs; E2—6 × 45 s 1 × 1 SSGs; BLa—blood lactate level; pH—blood pH; BE—base excess; HCO_3_^−^—blood bicarbonate; %HRmax—percentage of maximum heart rate; T0—before first SSG, T1—after first SSG; T2—after second SSG; T3—after third SSG; T4—after fourth SSG; T5—after fifth SSG; T6—after sixth SSG; T7—15 min after sixth SSG; T8—30 min after sixth SSG.

A significant interaction of the group and time effects was observed for the HCO_3_^−^ level, BE level, and %HRmax (Fig. 2). From T2 to T8, significantly lower HCO_3_^−^ levels were observed in group E1 than in group E2 (T2: *p* = 0.0390, d = 1.20; T3: *p* = 0.0148, d = 1.60; T4: *p* = 0.0124, d = 2.02; T5: *p* = 0.0136, d = 1.97; T6: *p* = 0.0007, d = 2.41; T7: *p* = 0.0000, d = 1.67; T8: *p* = 0.0022, d = 0.84). Except in T7 and T8, the course of the changes in the %HRmax was similar in both SSG groups. A significantly greater (T7: *p* = 0.0214, d = 1.01; T8: *p* = 0.0432, d = 0.87) 15 and 30 min post-exercise restitution %HRmax was observed in group E2 than in group E1. After the sixth SSG bout and 15 min restitution (T6: *p* = 0.0461, d = 0.34; T7: *p* = 0.0104, d = 1.87), significantly lower BE levels were found in group E1 than in group E2. No significant interactions for the group and time effects were found for the BLa level and pH (Fig. 2).

### Correlation between the ETL and ITL indicators

Table 5 shows the correlation between the ETL and ITL indicators. The statistical analysis showed a significant negative correlation between the %HRmax and the TD and TPL and a significant positive correlation between the HCO_3_^−^ level and the TD and TPL.

**Table 5:** Correlation between the average values of ETL indicators from all six SSGs and the values of ITL markers measured after the sixth game.

	%HRmax_T6	pH_T6	BE_T6	HCO_3_^−^_T6	BLa_T6
Total distance	** − 0.4997**	0.3333	0.2596	**0.6401**	0.0699
	***p* = 0.025**	*p* = 0.151	*p* = 0.269	***p* = 0.002**	*p* = 0.770
Total player load	** − 0.5993**	0.3099	0.2598	**0.5978**	0.0152
	***p* = 0.005**	*p* = 0.184	*p* = 0.269	***p* = 0.005**	*p* = 0.949
Player load per minute	− 0.1675	− 0.0346	− 0.0749	− 0.235	0.0935
	*p* = 0.480	*p* = 0.885	*p* = 0.754	*p* = 0.319	*p* = 0.695
Distance per minute	0.0011	− 0.0505	− 0.1427	− 0.4338	0.2017
	*p* = 0.996	*p* = 0.833	*p* = 0.548	*p* = 0.056	*p* = 0.394
Maximum velocity	− 0.1519	0.0971	0.0008	− 0.1811	0.2119
	*p* = 0.523	*p* = 0.684	*p* = 0.997	*p* = 0.445	*p* = 0.370

SSGs—small-sided games; BLa—blood lactate; pH—blood pH; BE—base excess; HCO_3_^−^—blood bicarbonate; %HRmax—percentage of maximum heart rate.

Significant values are in bold.

## Discussion

This study was designed to determine the influence of 1 × 1 SSGs with different bout durations (30 s vs. 45 s) but with the same work-to-rest ratio (1:4) on the ETL (GPS metrics) and ITL (BLa level, pH, BE level, HCO_3_^−^ level, and HR) and the relationship between these variables. The three main findings were as follows: (1) Extending the bout duration from 30 to 45 s increased the volume (TD and TPL) of effort and decreases the intensity of effort (TD per minute, TPL per minute, and Vmax); (2) extending the bout duration did not significantly affect the pH and BLa level; however, 30 s 1 × 1 SSGs yielded a greater decrease in the HCO_3_^−^ and BE levels and slowed down the restitution of the HR; and (3) a longer TD and a higher TPL resulted in weaker changes in the HR and HCO_3_^−^ level.

In line with previous research^18,61,62^, we recorded a higher effort intensity (TD per minute, PL per minute, and Vmax) but a lower training effort volume (TD and TPL) in shorter bouts—30 s SSGs. Köklü et al.^18^ used different SSG bout durations but the same overall time (6 × 2, 4 × 3, and 2 × 6 min) and found that extending the bout time decreased the SSG intensity (significantly longer distances covered in walking and shorter distances covered in moderate-intensity running in 2 × 6 min bout durations than in 6 × 2 min bout durations). A decrease in exercise intensity with an increase in bout duration (4 × 5, 2 × 10, and 1 × 20 min) was also observed by Alcântara et al.^62^, who stated that a shorter bout duration elicited a longer TD per minute and distance covered with > 80% peak speed in Carminatti's test. Since both Köklü et al.^18^ and Alcântara et al.^62^ used SSGs with different bout durations but the same total duration in their experiments, they observed an increased volume of efforts along with a shortened bout duration (longer TD in shorter bouts), contrary to our data. Similarly, Clemente et al.^63^ reported decreases in the TD and running distance and number of total accelerations and total decelerations when they compared between 3 × 6 and 6 × 3 min SSG formats. Conversely, Esqueda et al.^64^ arrived at opposite conclusions that long bout (LB; 3 × 6 min)-based SSGs seem to yield greater physical and physiological demands than do short bout (SB; 6 × 3 min)-based SSGs. They noted a longer TD and a larger number of accelerations of > 2 m s^−2^ and decelerations ≤ 2 m s^−2^ in LB-based SSGs than in SB-based SSGs.

One of the most common ITL markers is the HR. The use of HR monitoring during exercise is based on the linear relationship between the HR and the rate of oxygen consumption during steady-state exercise^65,66^. The HR can be used in many forms to monitor training load, including the %HRmax.

The mean %HRmax during the SSGs ranged from 86.7 to 90.8% in the current study, consistent with previous reports^19,31,37,61^. We did not observe any time effect for the %HRmax during all six bouts of SSGs in both groups. At rest and during the SSGs, we also did not observe any group effect for this indicator. A group effect for the %HRmax was observed in the post-exercise period: A significantly faster 15 and 30 min HR restitution was observed in group E2 than in group E1. We supposed that despite the greater volume of exercise noted in group E2, the faster recovery of the HR was influenced by the lower exercise intensity and longer duration of rest breaks (despite the same work-to-rest ratio). Clemente et al.^63^, who compared 5 × 5 SSGs in different time formats (3 × 6 and 6 × 3 min), did not also observe any time and group effects for the %HRmax. No group effect for the HR was demonstrated by Ade et al.^31^, who compared SSGs in speed endurance production (SEP) and speed endurance maintenance (SEM) formats (eight games lasting 30 and 60 s separated by 120 and 60 s of recovery with a work-to-rest ratio of 1:4 and 1:1, respectively). Fanchini et al.^17^, who compared 3 × 3 SSGs with different bout durations (three games lasting 2, 4, and 6 min), showed that the HR measured in the first set was significantly lower than that in the second and third sets, and the HR measured in 6 min SSGs was significantly lower than that in 4 min SSGs. Higher HRs in LBs of SSGs (LB: 3 × 6 min) than in SBs of SSGs (SB: 6 × 3 min) were measured by Esqueda et al.^64^. These authors stated that in SBs, the HR increased in the second and third bouts compared with that in the first bout and then decreased in the fourth, fifth, and sixth bouts compared with that in the second and third bouts. In addition, Köklü and Alemdaroğlu^67^ found that the %HRmax during the first bout was significantly lower than that during the other seven bouts in all formats of SSGs. The same authors^18^ reported that SB formats elicited significantly lower %HRmax responses than did LB formats, similar to the findings by Esqueda et al.^64^. Despite the differences in the results obtained in the above-mentioned experiments, our data show that in SSGs with SBs (30–45 s), the HR, unlike the ETL indicators, does not differentiate the exercise intensity, consistent with the findings by Ade et al.^31^. Our findings are also in line with the concerns expressed by other authors^68^ who believe that short-bout efforts (e.g., SB SSGs) involving a high anaerobic component that does not reach the steady-state phase may underestimate the HR response.

The second ITL indicator used in our study was the BLa level, which is extensively used as a parameter of exercise intensity in soccer^1,18,36,37^. The values measured in both groups after the fifth and sixth games exceeded 16 or even 17 mmol/L and are higher than previously reported data^17,18,31,37,61^. The values obtained herein indicate a very high activation of anaerobic metabolism and accumulation of BLa resulting from short rest breaks. Similar to the %HRmax, we did not observe any group effect for this indicator. In both studied groups, the BLa level after the first and second bouts was significantly lower than that after the other bouts, while that after the third bout was significantly lower than that after the fifth and sixth bouts. However, these results indicate that single 30 or 45 s loads are too short to cause an adequate BLa response. The BLa level after 15 and 30 min restitution in both groups was significantly lower than that after the sixth bout of SSGs but did not return to the pre-exercise level. Contrary to our research, Bekris et al.^37^ did not observe any differences between the BLa level measured after the second, fifth, and eighth bouts during 3 × 3 SSG training in 8 × 3 min format, which shows that longer (e.g., 3 min) efforts yield an adequate BLa response. A group effect was observed by Ade et al.^31^, who stated that SEP SSGs induce higher BLa levels than do SEM SSGs. Ade et al.^31^ suggested that SEM training requires a greater contribution from the aerobic energy system than does SEP training. A group effect was also observed by Köklü et al.^18^, who found that LB SSGs generated significantly lower BLa responses than did SB SSGs.

To date, there is no information available on the effects of SSG training on ABB. Few studies among soccer players have investigated the influence of simulated match plays^39,51^ or intermittent fitness tests^50^ on ABB indices. Only limited studies have shown the influence of successive bouts during intermittent exercises on acid–base homeostasis^69,70^. From a practical point of view, determination of exercise intensity based on the BLa level should be supplemented with ABB indicators. The excess H^+^ produced during exercise can be buffered by HCO_3_^−^, resulting in the formation of nonmetabolic CO_2_, which is removed during respiration^47,48^. Exercise-induced reduction in pH and disturbances in other ABB indicators, such as the BE or HCO_3_^−^ level, are one of the potential triggers of fatigue in soccer^1,45,46^. Indices such as the pH, HCO_3_^−^ level, and BE level, which are directly related to the BLa level, can and should be evaluated in the assessment of exercise intensity in soccer training. Expectedly, as with the BLa level, we observed a significant time effect for all ABB indicators in both groups. During SSG training, we noticed gradually worsening ABB disorders. The pH, HCO_3_^−^ level, and BE level measured after the first bout were significantly higher than those recorded after each subsequent bout. The values recorded after the fourth, fifth, and sixth games were significantly lower than those after the second game. Other authors also observed similar changes in the pH, HCO_3_^−^ level, and BE level in consecutive repetitions of high-intensity 10 (10 cycling sprints separated by 30 s recovery intervals) and 30 s (3 cycling sprints set at 90% of peak power, separated by 4 min recovery intervals) interval efforts^69,70^. In our study, stabilization of ABB disorders occurred after the fourth bout of SSG training. These changes indicate that single short (30 or 45 s) SSG bouts do not yield an adequate exercise intensity for metabolic responses. Among the measured ABB parameters, only the pH returned to the pre-exercise values after the 30 min restitution. This indicates that the buffering potential depleted during high-intensity SSG training requires a recovery period of more than 30 min. Similar to the BLa level, no significant group effect was found for the pH. From T2 to T8, significantly lower HCO_3_^−^ levels (by 2 mMol/L on average) were observed in group E1 than in group E2. These differences indicate that SSGs with shorter repetitions result in more severe metabolic acidosis than do SSGs with longer repetitions. Additionally, measurements of the BE level are also in line with this statement. After the sixth SSG bout and 15 min restitution, significantly lower BE levels were found in group E1 than in group E2. These results showed that the intergroup differences in the ETL indicators (i.e., TPL, TD, TPL per minute, TD per minute, and Vmax) were confirmed only with the change in the BE or HCO_3_^−^ level and not in the BLa level, which is commonly used to assess ITL in soccer training. Furthermore, our results showed that the significant changes in the ABB indicators were caused by more intense (GPS metrics), shorter-bout SSG training. This observation was also confirmed by the positive correlations found between the TPL and TD and the HCO_3_^−^ level. Moreover, a negative correlation was demonstrated between these GPS metrics and the %HRmax. These results suggest that extending the bout duration from 30 to 45 s increases the volume and decreases the intensity of exercise, thus reducing the body's physiological response.

Based on the present results on both BLa level and HR, it can be concluded that the use of these indicators is limited in differentiating ITL in SSG training with an SB duration (30–45 s). Therefore, Coutts et al.^29^ suggested that the RPE may be a more valid marker of global exercise intensity than the BLa level and HR. Further, Ade et al.^31^ emphasized that the HR and BLa should be used in addition to another monitoring parameter, such as the RPE or time–motion analysis data, to estimate the intensity of short-duration SSGs. Based on our findings, it seems justified to extend ITL monitoring to include blood biochemical indicators related to ABB.

Despite several important findings, some limitations are worth mentioning. The study was conducted among U18 players, which may not reflect actual changes, especially in younger age groups. As some authors suggest, it is reasonable to use not only the objective indicators we considered but also subjective indicators, such as the RPE, in ITL monitoring. Finally, for a more detailed ETL analysis, a wider range of GPS metrics, such as the speed zone, number of total accelerations and decelerations, and metabolic load, can be used. In the future, it seems reasonable to extend the analyses to include other age groups and SSG formats with a different duration and number of players.

## Conclusions

This study provides new information on monitoring training loads in SSG training with an SB duration. GPS metrics, such as the TD covered, TPL, TD covered per minute, TPL per minute, or Vmax, sensitively differentiate the ETL in SSG training with an SB duration. The HR and BLa level, widely used indicators of ITL in soccer, show limited diagnostic value in this type of training. It seems reasonable to extend ITL monitoring, particularly in SSG training considerably involving anaerobic metabolism, using ABB indicators, such as the HCO_3_^−^ and BE levels. Finally, extending the bout duration in SSG training from 30 to 45 s increases the volume of exercise and decreases the intensity of exercise, which reduce the physiological response among youth soccer players.

## Data Availability

The datasets generated during and/or analyzed during the current study are available from the corresponding author on reasonable request.
